# The Incidence and Clinical Characteristics of COVID-19 in Children With IBD During the Omicron Wave: A Single-Center Experience in China

**DOI:** 10.1155/grp/1868214

**Published:** 2025-01-16

**Authors:** Rui Li, Pei-Yu Chen, Hui-Wen Li, Lu Ren, Yang Cheng, Li-Ying Liu, Li-Juan Wei, Zi-Huan Zeng, Wan-Fu Xu, Si-Tang Gong, Lan-Lan Geng

**Affiliations:** Department of Gastroenterology, Guangzhou Women and Children's Medical Center, Guangzhou Medical University, Guangzhou, Guangdong Province, China

**Keywords:** COVID-19, cross-sectional study, disease course, incidence, inflammatory bowel disease, pediatric, risk factors

## Abstract

**Background and Aims:** The pandemic of coronavirus disease 2019 (COVID-19) had a major impact on the health of people worldwide, including the pediatric inflammatory bowel disease (PIBD) patients. As no study has investigated the susceptibility and disease course of COVID-19 in PIBD patients after the end of zero-COVID policy in China, we conducted a retrospective cross-sectional study in our center.

**Methods:** A cross-sectional survey enrolling PIBD patients has been completed by online survey, phone, and face-to-face assessment. The demographic data, epidemiological characteristics, clinical manifestations, treatment, and prognosis of the patients were analyzed.

**Results:** PIBD patients represented 55.45% (56/101) of SARS-CoV-2-positive cases between December 1st 2022 and January 31st 2023; 67.86% were male; the mean age was 11.15 ± 3.92 years old. Among the SARS-CoV-2-positive cases, three patients (5.36%) were asymptomatic, and 53 patients (94.64%) had mild symptoms. The main symptoms were fever (92.86%), cough (69.64%), nasal congestion or running nose (35.71%), and sore throat (33.93%). No severe case or deaths were reported. All patients recovered from COVID-19 symptoms within 1 week. We found no significant association between the type of inflammatory bowel disease (IBD) (Crohn's disease, ulcerative colitis, and unclassified) and SARS-CoV-2 infection rates, nor did we observe any correlation between different treatments and the risk of infection. Fifty-one patients were reported to be in close contact with persons confirmed with COVID-19 infection, and out of them, 36 patients test positive for SARS-CoV-2, which is significantly higher than that in patients without exposure to COVID-19 (70.59% vs. 33.33%, *p* = 0.002). A total of 10 patients were underweight, of which nine patients tested positive for COVID-19 (90% vs. 51.65%, *p* = 0.048). Meanwhile, unvaccinated patients were also found to be more susceptible to SARS-CoV-2 than vaccinated patients (70.97% vs. 48.48%, *p* = 0.049). The multivariable analysis showed that patients with moderate/severe activity of IBD were associated with an increased risk of SARS-CoV-2 infection (odds ratio (OR), 1.12; 95% confidence interval (CI), 1.13–8.33, *p* = 0.028).

**Conclusions:** The incidence of SARS-CoV-2 infection in our center of PIBD patients during the Omicron pandemic was 55.45%. No severity or death case was observed. The incidence was higher in underweight and unvaccinated IBD children. Patients with moderate/severe activity of IBD were at a higher risk of SARS-CoV-2 infection.

## 1. Introduction

The coronavirus disease 2019 (COVID-19), caused by the novel coronavirus SARS-CoV-2, is an acute infectious respiratory syndrome first reported in December 2019 [1]. It exponentially spread across all continents in just a few weeks and brought great challenges to global public health [2]. The World Health Organization (WHO) has defined five SARS-CoV-2 variants of concern (VoC), named Alpha, Beta, Gamma, Delta, and Omicron. Since its identification in November 2021, the Omicron variant has spread rapidly around the globe and caused large outbreaks in children and adolescents [3–6].

It has been reported that immunocompromised patients are at increased risk of severe illness and mortality from infection, and serious complications may occur in children, especially those with chronic and underlying conditions [7]. Characterized by immune-mediated chronic inflammation of the gastrointestinal tract, patients with inflammatory bowel disease (IBD) get increasing concern about the impact of COVID-19. Whether disease activity and biological treatment are risk factors for COVID-19 in IBD patients remains controversial; the majority of previous research suggested no difference in risk of COVID-19 in IBD patients when compared to that in the general population [6, 8–11]. The actual risk of infection or development of COVID-19 in these at-risk patients with IBD is not clear, especially as China's pandemic prevention and control measures have entered a new phase.

In the past few years, China has responded by implementing nonpharmaceutical interventions (NPIs) with a series of public health and social measures, so-called zero-COVID policy. After the announcement of “20 measures” in December 2022, the management of COVID-19 has been downgraded from Class A to Class B in accordance with the country's law on infectious disease prevention and treatment, meaning the end of zero-COVID policy and the entrance to a new phase of the COVID-19 response [12]. In China, the socioeconomic development is highly concentrated, usually in the eastern and southern regions. The huge population, high population density, increased rapid urbanization, and population mobility are also important to in the emergence and rapid spread of epidemic diseases [13, 14]. In this new phase of the pandemic, China is facing unprecedented challenges, so do the IBD children. With this in mind, we investigate the epidemiology, clinical characteristics, and outcomes of COVID-19 in pediatric inflammatory bowel disease (PIBD) patients. We conducted a cross-sectional study among the PIBD patients in our tertiary IBD center, in the first month following the transition of prevention policy; all IBD patients younger than 18 years old were included.

## 2. Materials and Methods

**Figure 1:**
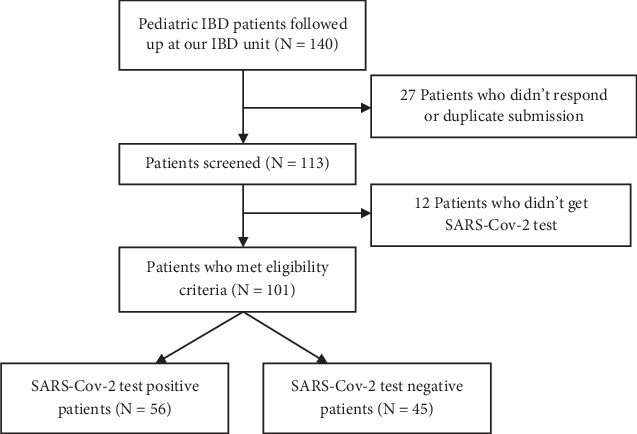
Flow diagram of the included patients.

To evaluate the impact of COVID-19 on PIBD patients, after the approval of the ethics committee of the Guangzhou Women and Children's Medical Center, we conducted a retrospective cross-sectional study among PIBD patients followed in our center (Guangzhou Women and Children's Medical Center Ethical Committee [2023], No. 181A01). All patients were individually contacted by online survey, phone, and face-to-face assessment between December 1st 2022 and January 31st 2023. Patients younger than 18 years old who were diagnosed with IBD were eligible for this study. The flow diagram of the included patients is demonstrated in Figure 1. An online survey was conducted in Chinese by the Questionnaire Star platform (https://www.wjx.cn). The questionnaire was filled in by the child's parents/caregivers, the information on clinical manifestation and follow-up assessments was reconfirmed by gastroenterologists at our institution. Additionally, we collected several key clinical indicators, including hemoglobin (HB), white blood cell (WBC) count, platelet (PLT) count, erythrocyte sedimentation rate (ESR), and albumin (ALB). The COVID-19 cases were confirmed according to the Diagnosis and Treatment Protocol for Novel Coronavirus Pneumonia (Trial Version 9) and WHO public health surveillance for COVID-19; the symptoms and severity of SARS-CoV-2 infection in children were classified as asymptomatic, mild, moderate and severe. Undernutrition of PIBD patients was described as thinness (low BMI-for-age), stunting (low height-for-age), and underweight (low weight-for-age) according to the WHO definition.

The survey documents the data on demographics, diseases, and conditions (including diagnosis, disease activity, and treatment) and information about COVID-19. Disease activity was calculated at the time of the last available clinical follow-up using pediatric Crohn's disease (CD) and ulcerative colitis (UC) activity indexes. All patients were asked to describe if they experienced subjective worsening of IBD symptoms and details for hospitalized patients during COVID-19. COVID-19 outcomes were classified as outpatient care, hospitalization, ventilator requirement, and death from COVID-19 or related complications.

Written informed consent was obtained from the parents of the included children during the primary data collection process. All data used in this study were again verified by gastroenterologists.

## 3. Statistical Analysis

Descriptive statistics were calculated for the basic demographic and clinical characteristics of the study population. Continuous variables were summarized as means and standard deviations and categorical variables as proportions. The differences between groups were compared using the Mann–Whitney *U* test, Kruskal–Wallis *H* test, and Student's *t*-test, as appropriate.

The chi-square test or Fisher's exact test was used to examine the differences between groups when the cell numbers were less than five.

The univariate logistic regression model was employed to investigate the association between relevant risk factors including age, sex, weight, height, IBD type, biological therapy, vaccination status, and disease activity. All stratification characteristics were included as categorical variables, and 95% confidence intervals (CIs) and Wald test results were reported. Factors with a *p* value less than 0.05 and those of known clinical relevance were included for further multivariable logistic regression analysis to obtain an adjusted odds ratio (OR) with 95% CIs. All statistical analyses were performed using a statistical software package (SPSS-20). Two-sided *p* values < 0.05 were considered statistically significant.

## 4. Results

### 4.1. Demographics and Characteristics of the IBD Children

A total of 101 questionnaires were analyzed; 56 of them test positive for SARS-CoV-2, which means the incidence of SARS-CoV-2 infection was 55.45%. Demographic and clinical characteristics of PIBD patients with SARS-CoV-2 test are summarized in Table 1. The mean age of PIBD patients was 11.08 years (±4.14). The majority of our patients were male, accounting for 65.35% of the total, which is consistent with the gender distribution observed in our SARS-CoV-2-infected patients, where males constituted 67.86%. The difference of COVID-19 incidence between male and female PIBD patients was not significant.

Patients with CD account for the majority of PIBD (91.09%), and most of the IBD patients were in clinical remission (51.49%) [15]. Among the 56 IBD patients who tested positive for SARS-CoV-2, 52 were CD, 1 was UC, and 3 were unclassified colitis. According to the Paris classification, we classified age at diagnosis as 0–< 10 years and 10–< 17 years; the rate of SARS-CoV-2 infection was about the same between two groups. Of all patients, the mean duration of disease was 2.54 years; the longest was 10 years.

As recorded in Table 1, the nutritional status of IBD children was divided into three subgroups based on the definition of WHO growth reference. Within the cohort, 14 (13.86%) patients were wasting, 6 (5.94%) patients were stunting, and 10 (9.9%) patients were underweight. Notably, nine out of ten underweight patients tested positive for SARS-CoV-2, indicating a statistically significant difference in COVID-19 incidence between underweight and nonunderweight PIBD patients (90% vs. 51.65%, *p* = 0.048). However, upon analyzing relevant clinical indicators such as HB, ALB, WBC, PLT, and ESR, we did not find any significant statistical differences. We hypothesize that this may be related to the majority of our patients being in a state of chronic malnutrition, which could potentially mask the impact of low body weight on these specific clinical parameters.

Of the included children, 61 patients (60.40%) were on biological treatment, 5 were on mesalazine, 6 were on thiopurines, and 3 were on steroids. Of the 61 patients on biological therapy, 40 were on infliximab, 20 were on adalimumab, and 1 was on ustekinumab. To be noticed, nine patients were in combo therapy (3 were on anti-TNF-*α* treatment together with thalidomide, 2 were on anti-TNF-*α* treatment and thiopurines, 1 was on thalidomide and mesalazine, 1 was on anti-TNF-*α* treatment together with mesalazine, 1 was on steroids and mesalazine, and 1 was on steroids and thiopurines). One patient was confirmed to have IL-10 receptor A (IL-10 RA) defect; one patient had comorbidity of immunodeficiency. There were no significant differences in the incidence of COVID-19 between different treatments.

Fifty-one patients were reported to be in close contact with persons confirmed with COVID-19 infection; out of them, 70.59% patients test positive for SARS-CoV-2, which is significantly higher than in patients without COVID-19 exposure (70.59% vs. 33.33%, *p* = 0.002).

### 4.2. Clinical Manifestation of SARS-CoV-2 Infection in Children With IBD

**Figure 2:**
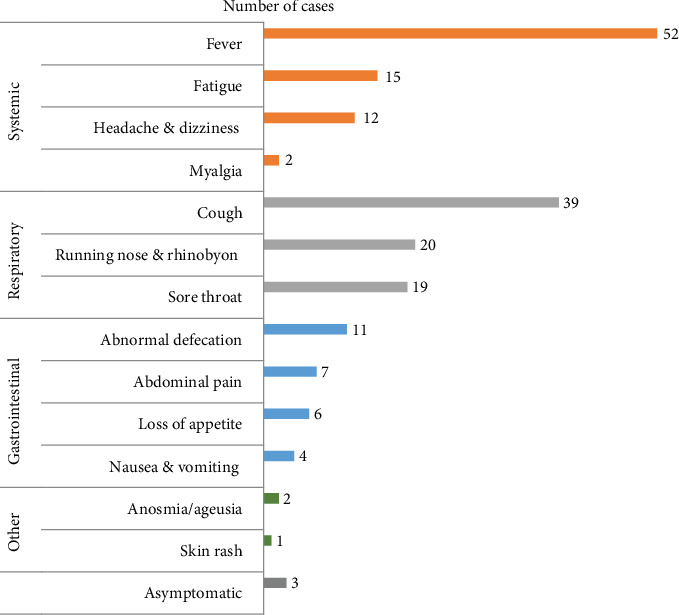
Clinical characteristics of IBD children with SARS-CoV-2 infection.

Among the 56 IBD patients who tested positive for SARS-CoV-2, 53 (94.64%) patients presented with mild symptoms and 3 (5.36%) were asymptomatic. No one was classified with moderate and severe COVID-19, and no patient died. A list of symptoms is summarized in Figure 2. The top five most common symptoms among symptomatic cases were fever (92.86%), cough (69.64%), rhinobyon and running nose (35.71%), sore throat (33.93%), and fatigue (26.79%); mean duration was 1.84 days. Because of the popularization of COVID-19 knowledge, 45 of our patients (80.36%) did not need medical consultation, 10 (17.86%) patients need outpatient care, and only 1 (1.78%) mild COVID-19 patient was hospitalized for 3 days.

### 4.3. Vaccination Status and Worsen of IBD Symptoms

Among the 101 children IBD patients involved, 97 patients were eligible for COVID-19 vaccination (aged ≥ 3 years), 66 (68.04%) of them received at least one dose of vaccination. The incidence of SARS-CoV-2 infection on unvaccinated patients and vaccinated patients was 70.97% and 48.48%, which showed a higher incidence of SARS-CoV-2 infection in unvaccinated patients (*p* = 0.049).

Among the 101 patients with IBD included in our cohort, 32 (14.6%) had spontaneously interrupted their therapy because of infection of SARS-CoV-2; all of them were treated with immunosuppressors and biologics.

### 4.4. Related Factors for SARS-CoV-2 Infection in PIBD Patients

We conducted an analysis to assess the impact of various factors on SARS-CoV-2 infection among our IBD patients, considering factors such as age, sex, weight, height, IBD type, biological therapy, vaccination status, and disease activity. Given the small number of patients with severe disease activity, we pooled this group with those having moderate disease activity for our calculations. The univariate analysis revealed that, within our cohort of IBD patients, the presence of moderate/severe disease activity was associated with a higher risk of SARS-CoV-2 infection (OR, 1.06; 95% CI, 1.13–7.37, *p* = 0.027). This association remained significant in the multivariable analysis after adjusting for age, gender, weight, and height (OR, 1.12; 95% CI, 1.13–8.33, *p* = 0.028).

## 5. Discussion

As described in our preprint, there is not much data about the incidence and the disease course of COVID-19 among IBD patients, especially in children and adolescents. We conduct the first population-based study of COVID-19 among PIBD patients and the first cross-sectional study after the end of the zero-COVID policy in China [15].

Although China has a large population of 1.3 billion, the incidence of IBD in China is significantly lower than that in the Western world, especially in children [16, 17]. The incidence was observed over 20/100,000 in those aged over 15 [18]. According to the *China Statistical Yearbook 2017*, children and adolescents aged < 14 accounted for 16.8% at the end of 2017 [19]. As the regional medical center of PIBD, our center has gathered more than 100 children with IBD from neighboring provinces, which is extremely difficult in China due to the lack of a nationwide registration system. Therefore, our study could be considered a snapshot of Chinese PIBD patients under the new control strategy, although we only had monocentric data. In our cohort, a total of 101 IBD children were included, the majority of them had CD (91.09%), and there was a slight predominance of males. Almost half of our patients were in clinical remission (51.49%). Fifty-six patients were found to have SARS-CoV-2 infection. The estimated incidence of SARS-CoV-2 infection in our study is 55.45%, which aligns with the range reported in previous studies involving general pediatric patients, spanning from 51.2% to 92.8, but much lower than that of the overall population in Guangzhou (> 85%) reported on the press conference on Jan 18 [20–22]. Even so, the incidence is significantly higher than that in other reports in other countries; it represents a breakthrough infection wave after the dynamic zero-COVID-19 strategy in China [23–25]. In addition, 11 patients were defined as highly suspected of COVID-19, which of whom presented influenza-like symptoms and closely contacted with confirmed cases, but did not have SARS-COV-2 test. The incidence of SARS-CoV-2 infection might be underestimated because of the suboptimal sensitivity of nasopharyngeal and oropharyngeal swabs for SARS-CoV-2 and the difficulty of getting tested [26]. Our patient population mostly lived in densely populated cities, and most of them were infected after the exposure to COVID-19, which facilitates the transmission of COVID-19.

Some studies indicate a higher risk of COVID-19 and mortality in patients with IBD, while some had the opposite conclusion [27–30]. In our cohort, the incidence of COVID-19 in IBD population is much lower than that in the general population [11], and a relatively small proportion of patients require hospitalization. No patient got severe COVID-19, and no deaths have been observed.

Studies show that young children under 5 years of age and patients with comorbidity have increased risk of getting COVID-19 [31]. However, in our study, 13 patients were younger than 5 years old; the infection rate of SARS-CoV-2 was about the same as that in other age group. But in patients with moderate/severe disease activity, the risk of SARS-CoV-2 infection was 1.12 times than that in the moderate/mild patients after adjusted analyze. There were reports showing that clinically active IBD may be a risk factor for severe COVID-19, particularly in younger patients, and the most important factors influencing outcomes were age and comorbidity [32–35]. But in our group, the number of severe COVID-19 outcomes were too few for stratified analyses. It can also be explained that Omicron variant infection in children/adolescents is associated with less severe disease than Delta variant infection [36].

Of the 101 patients included, 60.40% were on biologic therapy, and most were on anti-TNF-*α* therapy. We did not observe a significant statistical difference in the incidence of COVID-19 in patients treated with or without biologic therapy. Patients treated with biologics had a favorable outcome, which is consistent with former reports [8]. In this study population, no severe course of COVID-19 was observed, symptoms reported by all patients were mild (mostly fever and cough). Waggershauser et al. reported that TNF inhibitors and ustekinumab show a protective role in preventing respiratory tract infections among older age (> 49 years) [37], and Lichtenstein et al. also reported that patients who received biologics had a significantly milder course of disease [8]. In our observation, we did not identify any association between COVID-19 infection rates and risk of severe course and IBD-related treatment, which was consistent with previous reports [23, 38].

The most frequent symptom in our cohort is fever, followed by cough, nasal symptoms, and sore throat, which is in common with the study of de Souza et al. [39]. They described the clinical characteristics of 1124 cases of children with COVID-19; fever was the most prevalent symptom, followed by cough, nasal symptoms, diarrhea, nausea, vomiting, fatigue, and respiratory distress.

Among the included patients, 22 (21.78%) were undernutrition; 10 of them were underweight. Remarkably, our analysis showed that nearly all (90%) of the underweight children with IBD in our sample tested positive for SARS-CoV-2 infection. There are reports showing that patients with higher nutrition risk have worse outcome [40]. In a large multiethnic cohort study of adults hospitalized with COVID-19, they found that patients who were underweight and those with BMIs above the overweight range were more likely to be intubated or die [41]. These studies showed the association between underweight and poor outcomes of COVID-19 [42]. As in our study, we observe a significant high rate of SARS-CoV-2 infection in underweight PIBD patients, but all of them did not get severe course of COVID-19. Future studies should be performed between the body mass and outcomes of COVID-19.

Several preliminary studies have found a reduced risk of hospitalization and severe outcomes for Omicron relative to the Delta variant of SARS-CoV-2 [43]. By evaluating neutralizing antibody response in a comparator group of children, who were vaccinated with two doses or a single dose of mRNA vaccine, Tang et al. found that the largest reduction (25.2-fold) of neutralization titers in vaccinated children was observed against the Omicron variant [44]. However, importantly, all eight of the nine children who received two doses of mRNA vaccine still showed pseudovirion neutralization assay (PsVNA) 50 titers (for a sample dilution that resulted in 50% virus neutralization) > 1:20 against Omicron. The vaccine induced a much broader neutralizing antibody response against SARS-CoV-2 VOC in naive children compared with the natural immunity induced following SARS-CoV-2 infection. Studies showed that a moderate vaccine effectiveness after two doses of BNT162b2 or CoronaVac in children and adolescents and three doses of either BNT162b2 or CoronaVac provides substantial additional protection against severe COVID-19 [45, 46].

Studies have shown that COVID-19 vaccine effectiveness in IBD patients is comparable with that in non-IBD controls [47]. But several researches showed that anti-TNF-*α* treatment has attenuated response to vaccination against COVID-19. A multicenter, prospective, case–control study in the United Kingdom also indicated the prioritization of immunosuppressed groups for further vaccine booster dosing, particularly patients on anti-TNF-*α* and JAK inhibitors [45, 46, 48]. These studies underscored the importance of vaccination.

A systematic review and meta-regression study showed that vaccine efficacy or effectiveness against SARS-CoV-2 infection decreased from 1 to 6 months after full vaccination by 21.0 percentage points among people of all ages [49]. In our study, 66 patients got vaccinated, only seven patients got booster vaccination, and most of our patients got last vaccination for more than 6 months before the Omicron wave. Among the patients who were eligible for COVID-19 vaccination, a higher risk of SARS-CoV-2 infection was found in unvaccinated patients than that in vaccinated patients. This result shows that even over 6 months after vaccination, COVID-19 vaccine remained efficient against SARS-CoV-2 infection.

Some studies reported that a complication of gastrointestinal disease observed in children is multisystem inflammatory syndrome in children (MIS-C), which included abdominal pain, diarrhea, ongoing fever, cardiac dysfunction, and multiple organ failure [50]. We observed that 16 patients had gastrointestinal symptoms; no one had MIS-C. There were some studies suggesting that this imbalance in immune homeostasis especially in patients with severe COVID-19 has been linked to gastrointestinal symptoms such as diarrhea [51]. Various etiopathogenetic hypotheses have been advanced to explain the occurrence of diarrhea in COVID-19 patients, including loss in enterocyte absorption capability, microscopic mucosal inflammation damage, and an impaired function of ACE2, which leads to a downstream dysbiosis and metabolite imbalance [52–56]. As for our patients, there was no apparent aggravation of diarrhea compared to general patients.

Former research showed that patients who discontinued or delayed therapy with anti-TNF-*α* agents or other biologics had a higher rate of relapse [23, 57]. In our cohort, none of our patients modified the current treatment regimen, while 42.57% delayed or withheld it because of the epidemic. The main reasons of delay were the infection of COVID-19.

In this study, we performed the first population-based study of SARS-CoV-2 infection among PIBD patients in China, furthermore, the first study after the end of the zero-COVID policy. Second, we included 101 PIBD patients in our institution; the data records are detailed. Third, we observe a higher rate of SARS-CoV-2 infection in underweight PIBD patients, and ≥ 1 dose of vaccination against infection was efficient against SARS-CoV-2 infection. The presence of moderate/severe disease activity was associated with a higher risk of SARS-CoV-2 infection.

Nonetheless, some limitations must be taken into consideration. First, PIBD cases were included only in our institute; the small sample size of our cohort of patients prevents us from drawing any final conclusions on the risk factors for COVID-19 severity in the PIBD population. But now, we are working to establish a multicenter IBD database to share and track conditions of children with IBD in each center. Second, during the COVID-19 outbreak after the end of NPIs, a great number of COVID-19 patients caused the overrun of hospital. The lack of testing might underestimate the incidence of SARS-CoV-2 infection in IBD patients somewhat. Third, there was no severe COVID-19 case in our cohort that prevents us to identify risk factors related to the IBD demographic and treatment.

## 6. Conclusions

In conclusion, we first reported the results of clinical data and incidence of SARS-CoV-2 infection in Chinese PIBD patients. The incidence of COVID-19 infection in our IBD cohort was 55.45%, which is found to be lower than that in the general population. All patients had mild to no symptoms. We also observed a higher rate of SARS-CoV-2 infection in underweight or unvaccinated PIBD patients, which highlights the importance of nutrition management and vaccination in IBD children and adolescents. And patients with moderate/severe disease activity might be faced with an increased risk of SARS-CoV-2 infection. Further, a follow-up study is necessary to help us conduct better strategies for children with IBD.

## Figures and Tables

**Table 1:** Demographics and clinical characteristics of pediatric IBD population.

	**All (** **N** = 101**)**	**SARS-CoV-2 TEST**		***p***
		**Positive (** **N** = 56**)**	**Negative (** **N** = 45**)**	
Male, *n* (%)	66 (65.35)	38 (67.86)	28 (62.22)	NS
Age, year (±SD)	11.08 (±4.14)	11.15 (±3.92)	10.96 (±4.49)	NS
IBD classification, *n* (%)				NS
CD	92 (91.09)	52 (92.86)	40 (55.89)	
UC	4 (3.96)	1 (1.78)	3 (6.67)	
Unclassified	5 (4.95)	3 (5.36)	2 (4.44)	
Age at the time of IBD diagnosis, *n* (%)				NS
< 10 years	50 (49.50)	28 (50.00)	22 (48.89)	
10–17 years	51 (50.50)	28 (50.00)	23 (51.11)	
Undernutrition, *n* (%)	22 (21.78)	11 (19.64)	11 (24.44)	
Wasting	14 (13.86)	7 (12.50)	7 (15.56)	NS
Stunting	6 (5.94)	2 (3.57)	4 (8.89)	NS
Underweight	10 (9.90)	9 (16.07)	1 (2.22)	0.048
IBD therapy, *n* (%)				NS
Mesalazine	5 (4.95)	2 (3.57)	3 (6.67)	
Oral/parenteral steroids	3 (2.97)	3 (5.36)	0	
6MP	5 (4.95)	4 (7.14)	1 (2.22)	
Biological therapy	61 (60.40)	37 (66.07)	24 (53.33)	
Anti-TNF-*α*	60 (59.41)	36 (64.29)	24 (53.33)	
Adalimumab	20 (19.80)	15 (26.79)	5 (11.11)	
Infliximab	40 (39.60)	21 (37.50)	19 (42.22)	
IL-12/23 inhibitor (ustekinumab)	1 (0.99)	1 (1.79)	0	
Thalidomide	6 (5.94)	2 (3.57)	4 (8.89)	
Drug combination, *n* (%)	9 (8.91)	6 (10.71)	3 (6.67)	NS
Exposure to COVID-19, *n* (%)	51 (50.50)	36 (64.29)	15 (33.33)	0.002

Abbreviation: NS = no statistical significance.

## Data Availability

The datasets used and/or analyzed during the current study available from the corresponding author on reasonable request.
